# Biomimetic CO_2_ Capture Unlocked through Enzyme Mining: Discovery of a Highly Thermo- and Alkali-Stable Carbonic Anhydrase

**DOI:** 10.1021/acs.est.4c04291

**Published:** 2024-09-23

**Authors:** Konstantinos Rigkos, Georgios Filis, Io Antonopoulou, Ayanne de Oliveira Maciel, Pavlos Saridis, Dimitra Zarafeta, Georgios Skretas

**Affiliations:** † Institute of Chemical Biology, National Hellenic Research Foundation, Athens 11635, Greece; ‡ Department of Biological Applications and Technologies, University of Ioannina, Ioannina 45500, Greece; § Institute for Bio-Innovation, Biomedical Sciences Research Center “Alexander Fleming”, Vari 16672, Greece; ∥ Department of Informatics and Telecommunications, National and Kapodistrian University of Athens, Athens 16122, Greece; ⊥ Biochemical Process Engineering, Division of Chemical Engineering, Department of Civil, Environmental and Natural Resources Engineering, Luleå University of Technology, Luleå 97187, Sweden; # Faculty of Biology, National and Kapodistrian University of Athens, Athens 15772, Greece

**Keywords:** carbon capture, carbonic anhydrases, BioHPC, metagenomics, industrial biotechnology

## Abstract

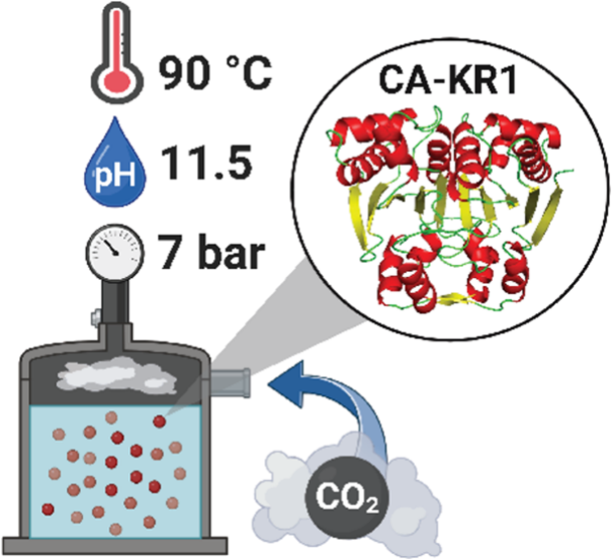

Taking immediate action to combat the urgent threat of CO_2_-driven global warming is crucial for ensuring a habitable planet. Decarbonizing the industrial sector requires implementing sustainable carbon-capture technologies, such as biomimetic hot potassium carbonate capture (BioHPC). BioHPC is superior to traditional amine-based strategies due to its eco-friendly nature. This innovative technology relies on robust carbonic anhydrases (CAs), enzymes that accelerate CO_2_ hydration and endure harsh industrial conditions like high temperature and alkalinity. Thus, the discovery of highly stable CAs is crucial for the BioHPC technology advancement. Through high-throughput bioinformatics analysis, we identified a highly thermo- and alkali-stable CA, termed CA-KR1, originating from a metagenomic sample collected at a hot spring in Kirishima, Japan. CA-KR1 demonstrates remarkable stability at high temperatures and pH, with a half-life of 24 h at 80 °C and retains activity and solubility even after 30 d in a 20% (w/v) K_2_CO_3_/pH 11.5 solution—a standard medium for HPC. In pressurized batch reactions, CA-KR1 enhanced CO_2_ absorption by >90% at 90 °C, 20% K_2_CO_3_, and 7 bar. To our knowledge, CA-KR1 constitutes the most resilient CA biocatalyst for efficient CO_2_ capture under HPC-relevant conditions, reported to date. CA-KR1 integration into industrial settings holds great promise in promoting efficient BioHPC, a potentially game-changing development for enhancing carbon-capture capacity toward industrial decarbonization.

## Abstract 2

Sustainable carbon-capture technologies are of vital environmental importance. The discovery of CA-KR1, a highly thermo- and alkali-stable carbonic anhydrase, signifies a leap forward toward the facilitation of biomimetic HPC carbon sequestration and industrial decarbonization.

## 1 Introduction

The 2021 Intergovernmental Panel on Climate Change (IPCC) Sixth Assessment report delivered a clear message: anthropogenic emissions, especially CO_2_, have caused an increase in the median temperature of our planet by 1.1 °C since preindustrial times.^1^ Projections for the future are alarming, as the average global temperature is expected to rise by 2–3 °C by the end of this century.^2^ Scientists have made severe warnings that if Earth’s temperature rises by more than 1.5 °C, detrimental effects for life and prosperity on our planet will likely occur.^3,4^ In response to this, the European Union has committed to reduce greenhouse gas emissions (GGEs) by approximately 40% by 2030 (compared to 1990 levels).^5,6^

A major driver of global warming is anthropogenic CO_2_ emissions,^7^ as their elevated levels result in infrared radiation and heat entrapment.^8^ Industrial CO_2_-containing flue gases are major contributors to this phenomenon. As such, industrial decarbonization through carbon capture, utilization, and storage technologies (CCUS) is one of the most promising approaches toward carbon neutrality and deceleration of global warming.^9−11^ CCUS include technologies designed to capture CO_2_ from industrial sources or direct air and convert it into various products or safely store it to prevent atmospheric accumulation.^9^

Among existing CCUS methods, hot potassium carbonate capture (HPC) has emerged as a highly promising, eco-friendly, and sustainable CO_2_ capture technology. HPC utilizes an aqueous K_2_CO_3_ solution as the sequestrating solvent: CO_2_-containing flue gases are passed through a pressurized absorption column containing the carbonate solution. CO_2_ dissolves and reacts with K_2_CO_3_ to form KHCO_3_, which acts as a temporary storage solution for CO_2_. Subsequently, in a separate desorption column with increased temperature and reduced pressure, the stored CO_2_ is recovered as a pure stream.^12^ An important process characteristic of HPC is that it operates at high temperatures (80–110 °C) and pressures (2–7 bar).^13,14^ Carbonate solutions offer distinct advantages when compared to conventional CO_2_ capturing solvents, like amines. Such advantages include minimal energy requirements for regeneration, noncorrosiveness, improved stability, and lack of toxicity, thus offering reduced operational costs and environmental friendliness.^13^ Carbonate solutions, however, exhibit a slower CO_2_ hydration reaction rate compared with amine-based capture. This reduced CO_2_ mass transfer in the liquid phase leads to higher capital costs, thus making HPC technologically inferior to current state-of-the-art methods. As an improved alternative, biomimetic HPC (BioHPC) utilizes specific CO_2_-sequestrating enzymes known as carbonic anhydrases (CAs, E.C. 4.2.1.1) to accelerate CO_2_ dissolution rates and promote the sustainable adoption of HPC.^15−17^

CAs are metalloenzymes requiring a bound metal ion for their structure and catalytic function.^18^ Their active site typically contains a zinc ion to facilitate the reversible hydration of CO_2_ to HCO_3_^–^ and H^+^ through metal-mediated nucleophilic attack.^19^ Without CA activity, this reaction proceeds slowly with a rate constant (*k*) of 3.5 × 10^–2^ s^–^.^1^^19^ CAs are among the most rapid enzymes in nature, with *k*_cat_ values up to 10^6^ s^–1^. They play crucial roles in maintaining cellular acid–base balance and facilitating CO_2_ transport in various biological processes, such as respiration and photosynthesis.^20^ The diversity of CAs is evident in eight different superfamilies, each clustered based on their structure and origin.^21^ Despite lacking significant structural and sequence similarities beyond their active sites, the consistent presence of CAs in biological systems suggests that nature has “redesigned” them multiple times, underscoring their significance.

As mentioned above, HPC operates at elevated temperature, pH, and salinity conditions, which often exceed the tolerance of conventional enzymes, including the vast majority of known CAs.^22−24^ Efforts to improve tolerance of already discovered CAs to industrially relevant conditions include protein engineering approaches like rational design and directed evolution, as well as CA-immobilization in novel materials such as metal–organic frameworks (MOFs) for integration into CA-catalyzed solvent absorption technologies.^25−27^ In addition to CA-catalyzed solvent absorption, advancements in CA-mediated membrane separation and CA-induced mineralization technologies further underscore the enzyme’s pivotal role in decarbonization strategies.^28^

A promising alternative approach for discovering new robust CAs is by mining the protein repertoire of extremophilic organisms, i.e., the ensemble of all of the proteins produced by microorganisms residing in environments with high temperature and alkalinity. Such organisms often encode and produce thermo- and alkali-stable proteins, which have evolved to sustain functionality under extreme conditions.^29,30^ More than 99% of extremophilic species, however, cannot be cultured using standard microbiology protocols^31,32^ and, thus, metagenomic analyses are typically employed for such purposes, either via experimental functional screening or through bioinformatic approaches.^33−37^

Bioinformatic approaches are exceptionally high-throughput and enable the screening of billions of potential enzyme-encoding genes present in extensive, metagenomic data sets. These data sets are increasingly becoming open-access, accelerating enzyme discovery.^38,39^ In this context, developing cutting-edge carbon-capture technologies, such as BioHPC, is accelerated by discovering resilient biocatalysts such as those discussed in this study. These technological advancements expedite the translation of CO_2_ capture research into industrial applications, supporting timely and effective decarbonization for crucial climate crisis mitigation.

## 2 Materials and Methods

All chemical reagents used in this work were purchased from AppliChem, and all molecular biology related products were purchased from New England Biolabs unless stated otherwise.

### 2.1 Bioinformatics Screening

Aiming to mine metagenomic data for the discovery of novel ultrastable CAs, metagenomic data from environments with registered temperature equal to or above 80 °C were sourced from the SRA and the MG-RAST server. The RUN accessions from the SRA and the metagenome identifiers from the MG-RAST server for these samples were collected. The SRA Toolkit from the National Center for Biotechnology Information (NCBI)^40,41^ was used to download the SRA file related to each RUN accession, validate its integrity, and convert it to its corresponding compressed FASTQ file(s). One file in FASTA format was downloaded for each MG-RAST metagenome identifier from the MG-RAST Web server that contained contigs, which were based on assembled reads. The FASTQ or FASTA file(s) of each sample was/were given as input to a pipeline of various analysis processes, which gave as output certain files that included the annotated putative proteins of interest. The data of the input FASTQ file(s) were initially analyzed by FastQC,^42^ which performs several quality control checks. The FASTQ file(s) was/were then processed by BBDuk,^43^ a tool for filtering or trimming reads for adapters and contaminants using *k*-mers. The next step was to align and assemble the processed reads into contigs based on MEGAHIT.^44^ MEGAHIT is an NGS de novo assembler for assembling large and complex metagenomic data in a time-efficient and cost-efficient manner. The contigs formed based on the input FASTQ file(s) or the contigs present in the input FASTA file (for one sample) were then provided as input to the gene prediction algorithm FragGeneScan.^45^ FragGeneScan has been proven to perform adequately on data of a metagenomic origin. The putative proteins encoded by the predicted genes were also provided by FragGeneScan. CD-HIT^46,47^ was then applied to the putative proteins with the goal of reducing their redundancy. Based on the “hmmscan” utility of HMMER,^48,49^ each putative protein was scanned against the SCAPs, and only those with at least one hit were processed further. These proteins formed the first set of proteins that could be further annotated. Proteins with no hits against the SCAPs were screened against the fnr database with BLASTP. Based on this screening, putative proteins, which had a hit with an *e*-value equal to or less than 1 × 10^–50^, were identified. These proteins formed the second set of proteins to be further annotated. The analysis based on the fnr database did not take place for every sample analyzed by the pipeline; thus, for several samples, only one set of proteins was formed for further annotation. In every case, each of the two sets of proteins followed the same analysis and annotation processes for the rest of the pipeline. At this stage, the “hmmscan” utility of HMMER was used to screen the proteins against all of the profiles of the Pfam database. The next analysis process was performed by Phobius.^50^ Phobius is a combined transmembrane topology and signal peptide predictor. Furthermore, BLASTP was used to scan the putative proteins against the Swiss-Prot protein sequence database of UniProtKB.^51−53^ From the SCAPs, 3 profiles, “Carb_anhydrase,” “Pro_CA,” and “CsoSCA,” were determined to be of the highest interest for the identification of putative CAs. Specific putative proteins were selected and further validated and annotated to curate a set of 10 proteins, which would later be tested experimentally. Each of the selected putative proteins was screened against the nr database, and its corresponding gene was screened for start codons (ATG, GTG, TTG) and stop codons (TAA, TGA, TAG). The length of each putative protein was also compared to the average length of the proteins in the CA family, and the presence or absence of signal peptides and transmembrane regions was also evaluated. In addition, the final set of the 10 selected putative proteins was examined based on CD-HIT to determine the existence of pairs of proteins with sequence identity equal to or higher than 90% to avoid selecting highly similar proteins. Furthermore, each protein from the final set of 10 proteins was screened against the motifs “CxDxR” and “HxxC,” which are related to the active site of the family of β-CAs.

### 2.2 Gene Cloning and Protein Purification

The corresponding gene sequences of the selected putative proteins were codon-optimized for efficient expression in *Escherichia coli*, and nine of them were successfully synthesized (IDT) to contain a hexahistidine-coding sequence at the 3′ terminus and *NcoI/XhoI* restriction sites at 5′ and at 3′ termini, respectively. Gene cloning was performed into the pET-28a(+) plasmid vectors using the aforementioned restriction sites, generating nine recombinant pET-caKRX_(1–9)_ plasmids. In this work, the pET-caKR1 plasmid, carrying the *ca-KR1* gene encoding the CA-KR1 protein, is discussed. *E. coli* Origami 2(DE3) (Novagen) chemically competent cells were transformed with the recombinant plasmid pET-caKR1. The resulting strain, Origami 2(DE3) pET-caKR1, was grown in LB broth media supplemented with Kanamycin (50 μg/mL) at 37 °C and 220 rpm until OD_600_ = 0.5–0.8. At this point, recombinant protein production was induced by the addition of isopropyl-β-d-thiogalactopyranoside (IPTG) at a final concentration of 0.2 mM. The culture was also supplemented with 0.5 mM ZnCl_2_, as CA-KR1 is a metalloenzyme. After 16 h of incubation, the cells were harvested with centrifugation at 6000*g*, 4 °C for 15 min. Cell pellets were carefully resuspended in 25 mM Tris–HCl/100 mM NaCl/10 mM imidazole (to prevent nonselective binding to the resin during IMAC chromatography), pH 8.3 buffer by mild manual stirring on ice. The cells were lysed with the use of a cell disruptor (Constant Systems, CF2 model) working at a pressure of 30 KPSI. The resulting lysate was subsequently fractionated by centrifugation at 47,000*g*, 4 °C for 45 min, and the supernatant was loaded on an immobilized metal affinity chromatography (IMAC) purification column. The buffers used were 25 mM Tris–HCl/100 mM NaCl/10 mM imidazole, pH 8.3 (for column equilibration), 25 mM Tris–HCl/100 mM NaCl/25 mM imidazole, pH 8.3 (for washing) and 25 mM Tris–HCl/100 mM NaCl/250 mM imidazole, pH 8.3 (for the elution of the protein).^54,55^ The purity of the eluted protein was evaluated by SDS-PAGE along with Coomassie Blue gel staining. The recombinant CA-KR1 was further purified by size exclusion chromatography (SEC), using a ÄKTA pure 25 system (GE Healthcare Lifesciences) with a HiLoad 16/600 Superdex 75 pg column and 25 mM Tris–HCl, 100 mM NaCl, and pH 8.3 buffer (Figure S2).

### 2.3 Carbonic Anhydrase Activity Assays and Kinetic Studies

After the successful purification of the CA-KR1 protein, its potential CA catalytic activity was assayed by employing a modification of the Wilbur–Anderson assay.^56^ For the preparation of the reaction, fully saturated CO_2_ water was prepared by bubbling pure CO_2_ gas in 200 mL of distilled, ice-cold H_2_O for at least 30 min. The reaction was initiated by the addition of 10 μL of CA-KR1 solution in 100 μL of 100 mM Tris–HCl, 0.2 mM Phenol red, pH 8.3 buffer, followed by immediate addition of 200 μL of CO_2_-saturated d.H_2_O. The final concentration of the enzyme in the reaction was 340 nM (6.5 μg/mL). For the control reaction, 10 μL of the same buffer, without the addition of the enzyme, was used instead. All reagents used were ice-cold, and multiple measurements were performed in parallel in 96 well plates, using a multichannel pipette for simultaneous mixing of all samples due to the time sensitivity of the reaction. The time required for the pH change from 8.3 to 6.3 was measured through the change in color of phenol red from red to yellow, directly reflecting the proton release during CO_2_ hydration for both catalyzed and uncatalyzed reactions.

In order to cross-verify the CA activity of CA-KR1, the protein was subjected to protonography with a few modifications of the original protocol.^57^ The protein sample was mixed with native loading dye (without SDS, mercaptoethanol, or DTT), loaded in duplicate on a 15% polyacrylamide SDS gel next to a protein standard sample, and run at 180 V for 45 min. Afterward, the gel was vertically split into two parts: the first one contained the protein standard alongside one of the two identical CA-KR1 lanes and stained with Coomassie blue stain, while the other half containing the other CA-KR1 lain was subjected to protonography to identify CA activity.

It is noteworthy that while some carbonic anhydrases (CAs) display side esterase activity,^54^ enabling their biochemical characterization using *p*-nitrophenyl conjugated fatty acid substrates, CA-KR1 did not show the ability to hydrolyze p-nitrophenyl acetate (pNPA). This lack of activity was consistent across various pNPA concentrations and temperatures ranging from 40 to 80 °C. Consequently, this study employed exclusively CO_2_ hydration assays to characterize the novel CA, thereby directly assessing the enzyme’s activity on its natural substrate.

For the determination of CA-KR1 kinetic parameters, a stopped-flow spectrophotometric method was employed using the stopped-flow device (SFA-20 Rapid Kinetics Accessory) (TgK Scientific) equipped with two 2.5 mL Kloehn drive syringes. CO_2_-saturated water (34 mM) was prepared by continuous bubbling of pure CO_2_ gas in 200 mL of distilled H_2_O at 25 °C for 1 h. All of the experiments were performed at 25 °C starting by diluting 50 μL of purified CA-KR1 (in 25 mM Tris–HCl, 100 mM NaCl, pH 8.3 buffer) (catalyzed reaction) or nonenzyme containing buffer (uncatalyzed reaction) in 3 mL of 20 mM Tris–HCl, 20 mM NaCl (to maintain constant ionic strength), and 0.2 mM phenol red pH 8.3, which were then loaded into a stopped-flow device drive syringe and secured. The other drive syringe was loaded with CO_2_-saturated d.H_2_O of concentration ranging from 6.8 to 34 mM. The reagents of the two syringes were rapidly mixed at a 1:1 ratio, and the absorbance change at 557 nm was recorded photometrically using a SPECTROstar Nano (BMG LABTECH) plate reader.^55,58,59^ The CA-KR1 concentration in the final reaction was 350 nM (6.7 μg/mL). The initial rates of absorbance change were recorded following the first 10–20 s of the reaction, and the uncatalyzed rates were subtracted from the catalyzed ones. Finally, the actual enzymatic CO_2_ conversion rates were calculated by multiplication of the subtracted absorbance rates with the *Q* buffering factor as previously described (eq S1).^60^ All measurements were performed in a minimum of two independent experiments of three technical replicates. The data were fitted in the Michaelis–Menten equation and analyzed using GraphPad Prism 9 software to calculate the kinetic constants.

### 2.4 Thermostability and Alkali-Stability Studies

The thermostability of the CA-KR1 enzyme was studied by measuring the residual (%) CO_2_ hydratase activity after prolonged incubation at different temperatures and for varying time periods. It is important to note that assays involving gaseous substrates such as CO_2_ tend to produce significant data deviations, especially when time measurements are taken by observation, a practice that introduces bias in the perception of color. Consequently, the method selected to eliminate such deviations was stopped-flow spectrophotometry. For each measurement, 75 μL of SEC purified CA-KR1 was initially mixed with 3 mL of 50 mM Tris-SO_4_ and 0.1 mM phenol red pH 8.3 and loaded in one of the reservoir syringes. The other syringe was filled with CO_2_-saturated water (continuous bubbling on ice for 1 h), and the contents of the two reservoirs were mixed instantaneously at a 1:1 volume ratio in the stopped-flow cell. The final concentration of the enzyme in the reaction was 1.28–3.84 μM (24.4–73.1 μg/mL). The time required for the absorbance at 570 nm to drop from the maximum (pH 8.3) to the value corresponding to pH 6.3 was recorded photometrically for both catalyzed and uncatalyzed reaction, and the activity was calculated in WA units, as described previously (eq S2).^61^ CA-KR1 aliquots were incubated at 80 and 90 °C for time periods ranging from 1–24 h, and the residual activity was calculated (eq S3). All measurements were performed at a minimum of 5 independent experiments (starting from protein overexpression) of a minimum of three technical triplicates.

The alkali-stability of CA-KR1 was studied in an application-realistic concentration of carbonates (20% K_2_CO_3_ (w/v), pH 11.5). For this purpose, purified CA-KR1 was mixed in a 1:1 ratio with 40% K_2_CO_3_ aqueous solution and incubated at room temperature for time periods varying from 1 to 30 d. The final concentration of the enzyme was 36.8–89.3 μM (0.7–1.7 mg/mL). At the end of each incubation period, the solution was centrifuged at 22,000*g*, 4 °C for 15 min to remove the denatured protein. The stability of the CA-KR1 was evaluated by measuring the residual concentration of soluble enzyme after incubation (eq S4). Upon measurements, all samples were also subjected to native PAGE, followed by Coomassie staining and Native Protonography (refer to Section 2.3) to correlate residual solubility with CA activity. All measurements were performed at a minimum of 3 independent experiments (starting from protein overexpression) of a minimum of three technical triplicates.

### 2.5 Enzyme-Promoted CO_2_ Capture in Pressurized Batch Reactor Study

A high-pressure bioreactor was designed and commissioned at Luleå University of Technology (Luleå, Sweden). The vessel was constructed using a 304L-HDF4 Stainless Steel Double Ended DOT-Compliant Sample Cylinder. The top convex portion of the reactor was connected to two SS tubing ports: one for feeding and the other for introducing gas. Additionally, a port was situated at the bottom of the reactor for liquid drainage. The system was equipped with a precise digital pressure gauge (DG-10, WIKA Alexander Wiegand SE & Co.KG) to monitor the pressure. The total and working volume of the reactor was 150/50 mL. Before initiating the experiment, the reactor underwent three rounds of cleaning with distilled water. Initially, 50 mL of 20% (w/v) K_2_CO_3_ was introduced into the reactor, which was then immersed in a water bath to maintain the desired temperature (20–90 °C). Once the system temperature stabilized, the pressure within the system was recorded, and the reactor was pressurized to 7 bar using a synthetic gas mixture (20:80 v/v CO_2_: N_2_, Linde Gas, Sweden). The pressure drop was recorded over time for 210 min. Enzyme-promoted reactions included the addition of 700 μL of purified CA-KR1, corresponding to an enzyme load of 18.7 ± 3.1 mg of enzyme per L of carbonate solution. All reactions were carried out in duplicates. The calculated productivity (expressed as the total amount of absorbed CO_2_ per L per min of reaction until the plateau phase), CO_2_ removal efficiency, and initial absorption rate were based on the ideal gas assumption and the fact that only CO_2_ is absorbed by the medium.

## 3 Results and Discussion

### 3.1 High-Throughput Metagenomic Analysis for the Identification of Putative CO_2_-Sequestrating CAs

Aiming to identify robust CAs with inherent stability against harsh conditions suitable for industrial CO_2_ capture applications, a custom bioinformatics pipeline was developed, which was in part based on the web-based application ANASTASIA.^62^ The purpose of this pipeline was to mine metagenomic data originating from extreme environments to identify protein sequences of putative extremophilic CAs. Toward this goal, 24 RUN accessions from the SRA and 4 metagenome identifiers from the MG-RAST server were retrieved from environmental samples with registered sampling temperature equal to or above 80 °C. To discover CAs that can be implemented in industrial CO_2_ capture pipelines with high pH and high-temperature solvents, such as HPC, metagenomic samples originating from high temperature and alkaline pH environments were targeted. This led to the selection of three metagenomic data sets (RUN accessions: DRR163688, SRR3961740, and SRR14762249). According to the analysis of these three data sets, 100,364 protein coding regions were predicted. In addition, several Pfam^63^ Hidden Markov Model profiles (pHMMs) related to CA domains were selected, and a set of CA profiles (SCAPs) was formed. The Pfam accessions of the latest versions of these profiles, available at the time of the analysis, were “PF00194.23”, “PF00484.21”, “PF00101.22”, “PF08936.12”, “PF00016.22”, “PF02788.18”, “PF00936.21”, and “PF03319.15” with the corresponding names of “Carb_anhydrase”, “Pro_CA”, “RuBisCO_small”, “CsoSCA”, “RuBisCO_large”, “RuBisCO_large_*N*”, “BMC” and “EutN_Ccml.” Among the predicted protein coding regions, two putative proteins were found to contain at least one “Carb_anhydrase” domain, 18 putative proteins were found to contain at least one “Pro_CA” domain, and one putative protein was found to contain at least one “CsoSCA” domain. Furthermore, 76 putative proteins were found not to have any domain of the SCAPs but at least one hit against a filtered version of the BLAST database “nr” (“fnr” database) with e-value equal to or lower than 1 × 10^–50^.^64^ The fnr database was the result of retaining the proteins of the nr database, each of which proteins included in its header the phrase “carbonic anhydrase” or “carbonic dehydratase” or “ca”. Therefore, it was possible to screen the putative proteins with the “blastp” functionality (BLASTP) of the BLAST+ applications^65,66^ against the fnr database much faster than to screen them against the nr database. Based on the complete annotation of the putative proteins, ten of them were selected, with no pair of these ten putative proteins having sequence identity equal to or higher than 90%. Following the above methodology, in this work, 2,234,568 protein coding regions were bioinformatically interrogated, out of which 702 were identified as putative CAs. Out of these, 368 sequences contained at least one of the SCAPs. Selecting the sequences that originated from metagenomic data corresponding to sampling conditions of alkaline pH and temperature ≥90 °C, a list of 31 putative thermostable and alkali-stable CAs was generated, which contained at least one of the SCAPs. The gene sequences of ten putative proteins, including *ca-kr1*, were eventually codon-optimized for recombinant production in *E. coli* and synthesized to be further studied. Our pipeline identified one domain within the CA-KR1 sequence, the Pro_CA domain (accession: PF00484.21) and did not predict the presence of a signal peptide or a transmembrane region. CA-KR1 was predicted to comprise 168 amino acids with a molecular weight of 19 kDa. The closest hit (with the lowest e-value) of CA-KR1 against the Swiss-Prot protein database had 41.9% identity and 52% query coverage and is a known CA enzyme from *Mycolicibacterium smegmatis* (UniProt accession number A0R5660). The closest hit (with the lowest e-value) in the nr database originates from *Pyrobaculum aerophilum* (accession WP_116420447.1) (94.6% identity, 100% query coverage), indicating that CA-KR1 may originate from the *Pyrobaculum* species. Both motifs, “CxDxR” and “HxxC,” related to the active site of the β-CAs family, were identified in CA-KR1, indicating that CA-KR1 belongs to the β family of CAs.^67−69^ The bioinformatics pipeline developed in this study to uncover novel carbonic anhydrases (CAs) from metagenomic data is currently being translated into a command line and an open-access online tool named *ProteoSeeker* (G. Filis et al., unpublished).

### 3.2 Recombinant Production and CA Activity Detection of CA-KR1

The *ca-KR1* gene, codon-optimized for expression in *E. coli*, was cloned into the expression vector pET-28a(+) to generate the recombinant plasmid pET-caKR1. pET-caKR1 was then used to transform the *E. coli* strain of Origami 2 (DE3), and CA-KR1 production was carried out at 37 °C upon addition of isopropyl-β-d-thiogalactopyranoside (IPTG) in shaking flask liquid cultures resulting in a production yield of 6.5 ± 0.5 mg of recombinant protein per L of liquid culture. After production, the recombinant enzyme was initially purified using immobilized metal ion affinity chromatography (IMAC). To test for potential CA activity, the purified recombinant protein was subjected to a colorimetric activity assay. The assay monitors CO_2_ hydration by detecting color changes of the pH indicator phenol red, which is triggered by a decrease in pH due to proton production upon biocatalytic conversion. The introduction of CA-KR1 led to a significantly accelerated color transition from red to yellow compared with the color change time of the control, demonstrating the enzymatic facilitation of CO_2_ hydration (Figure S1A). To further test this indication, protonography of CA-KR1 further purified via size exclusion chromatography (SEC) was performed in parallel on a semidenaturing SDS-PAGE, followed by Coomassie staining (Figure S1C). CA activity was additionally detected in-gel in this assay by observation of the characteristic color change of the pH indicator bromothymol blue from blue to yellow, indicating a local decrease in pH due to the release of protons in the CO_2_ hydration reaction after immersion of the gel in CO_2_-saturated water (Figure S1B). Protonography revealed distinct CA activity zones within the gel. These results indicate that CA-KR1 is an active, dimeric CA, as confirmed by the SEC purification profile, which revealed a dimeric oligomerization state of the protein (Figure S2).

### 3.3 CA-KR1 Thermostability

**Figure 1:**
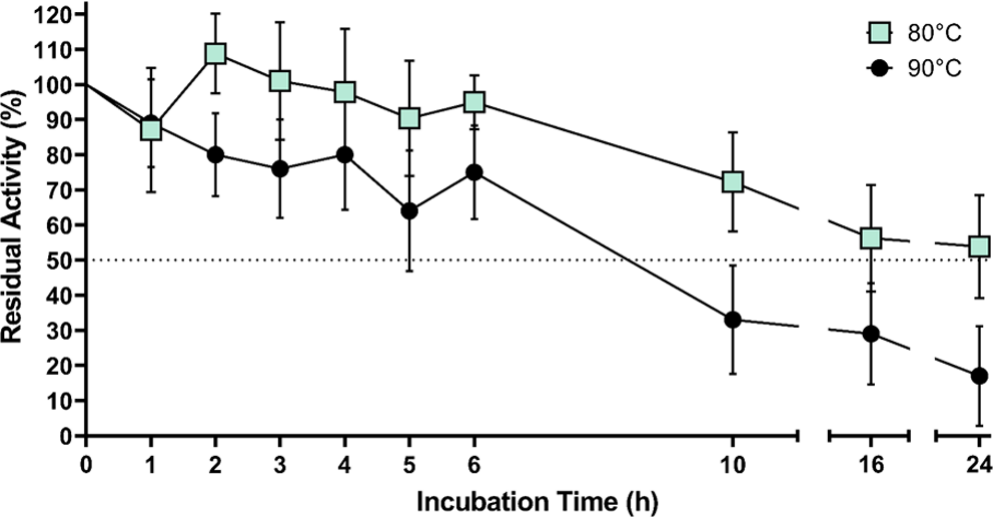
CA-KR1 thermostability. CA-KR1 thermostability was evaluated by measurements of residual hydratase activity after exposure at 80 and 90 °C for up to 24 h. The presented data correspond to the mean value of a minimum of five independent experiments, each conducted in replica triplicates. The error bars indicate the standard deviation of the mean values.

One of the most crucial characteristics a CA must exhibit to be considered a candidate biocatalyst for CO_2_ sequestration is thermal stability. This is because, during industrial carbon-capture processes, temperatures above 80 °C are used. CA-KR1 thermostability was evaluated by incubating the enzyme at 80 and 90 °C for up to 24 h and measuring its residual catalytic activity using the standard stopped-flow protocol (see Section 2.4). As shown in Figure 1, CA-KR1 exhibits a catalytic half-life of 24 h when incubated at 80 °C and retains approximately 75% of its initial activity after 6 h of incubation at 90 °C. Interestingly, CA-KR1 seems to exhibit a “thermal activation” profile after incubation at 80 °C for 2 h, which indicates a near-optimal temperature of action under these experimental conditions (*T* = 80 °C, pH 8.3). While the thermal activation of thermostable enzymes is well-documented, the precise mechanism behind it remains elusive.^70−72^ Recent studies suggest that thermal incubation of these enzymes induces motions that propagate through the protein structure, repositioning active sites to enhance catalytic efficiency.^73,74^ While this could potentially explain the thermal activation observed in CA-KR1, extensive structural and biophysical studies are required to elucidate the exact mechanism. Only one natural thermostable β-CA, namely, Cab from the *Methanobacterium thermoautotrophicum* has been reported to date, exhibiting ∼72% of residual activity after 15 min of incubation at 80 °C and ∼27% of residual activity after incubation at 90 °C for the same time period.^75^ The most widely studied CAs for potential CO_2_ sequestration are the SspCA^54^ and TaCA^55^ enzymes, both belonging to the α-CA family and sharing over 500 literature references in Google Scholar. SspCA has been reported as a benchmark enzyme for industrial CO_2_ capture, as it exhibits high thermostability in temperatures relevant to the application. Specifically, SspCA retains ∼76 and ∼71% of its initial activity after 3 h of incubation at 80 and 90 °C, respectively.^54^ In comparison, CA-KR1 exhibits 100 and 77% residual activity when tested under the same conditions. In the case of TaCA, the oxidized enzyme oTaCA is reported to be the most thermostable conformational state of the enzyme. oTaCA has been reported to maintain ∼65 and ∼35% of its activity after 1 h of incubation at 80 and 90 °C, respectively,^55^ while at the same conditions, CA-KR1 exhibits 87 and 89% of residual activity, respectively. Furthermore, TaCA has been recently referred to as the most thermostable CA ever discovered based on the fact that it exhibits a half-life of 77 d at 60 °C,^76^ although this temperature is not optimum for CO_2_ capture. Numerous studies have applied protein engineering strategies to optimize CAs and enhance their thermostability. In fact, the most recently discovered thermostable CA is a mutant of SspCA, named K100G, acquired through rational design, which retains 30% of its activity after 1 h of incubation at 85 °C.^77^ In comparison, CA-KR1 shows superior thermostability as it exhibits 89% of residual activity after 1 h of incubation at the highest tested temperature of 90 °C. TaCA(N140G), another variant engineered through rational protein design aiming to increase thermostability, has an improved 203-d half-life at 60 °C, but experiments showing 100% residual activity at 80 and 90 °C have only been conducted for up to 1 h.^78^ In comparison, CA-KR1 retains nearly 100% of its initial activity at 80 °C for up to 3 h, while at 90 °C after 1 h of incubation, its residual activity is 89%. Very few discovery studies test new CAs in realistic CO_2_-capture setups. Such experiments, when studying biocatalysts having unknown biochemical profile, are really challenging as the enzyme concentration requirements range from 0.2–2 g/L^79^ in large-volume bioreactors, making it unsustainable for lab-scale experiments. One of these studies reports the testing of TaCA derivatives acquired via directed evolution in a scaled CO_2_ absorption–desorption setup in the presence of solvents, such as amines or carbonates, and the measuring of the actual Δ*C*_CO_2__ between the inlet and outlet of the unit.^79^ Specifically, the triple mutant TaCA (S8R/G9P/E22P) presented 73% of residual activity (in contrast to 21% of the wild type) after 24 h in 20% w/v K_2_CO_3_. CA-KR1, although tested in a lab setup, after 24 h of incubation at 90 °C, maintains ∼18% of its initial activity, which is close to that of the wild-type TaCA in the aforementioned study at 85 °C.

A concise summary of the thermostability of known carbonic anhydrases from various studies can be found in Table S1.

Although not all available CA biochemical characterization data can be directly compared due to the different scale and experimental methods being used, the above comparison strongly highlights the potential of CA-KR1 as a leading CO_2_-sequestration biocatalyst based on its inherent thermostability and overall alkali-stability (see Section 3.4). Overall, these findings rank CA-KR1 as the most thermostable β-CA discovered to date and probably, to the extent of our current knowledge, among the most thermostable CAs reported so far.

### 3.4 CA-KR1 Stability in Carbonate Solution

**Figure 2:**
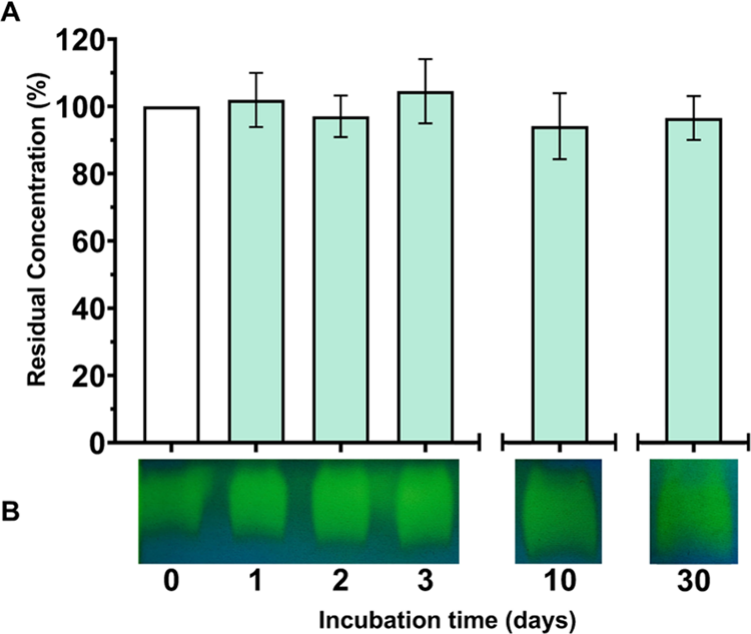
CA-KR1 stability in 20% K_2_CO_3_. (A) Alkali-stability of CA-KR1 was evaluated through the measurement of residual solubility after incubation of the enzyme in aqueous 20% (w/v) K_2_CO_3_ aqueous solution for different time periods spanning up to 30 d. The incubated enzyme preparation was centrifuged to remove denatured protein, and the remaining soluble protein concentration was measured photometrically. The measurements of residual soluble protein were performed in two independent experiments of technical triplicate and expressed as the mean average with standard deviation. (B) Protonography assay. The incubated enzyme was subjected to protonography analysis to visualize the CA activity.

The state-of-the-art biomimetic CO_2_ capture technologies that lead the carbon-capture field use carbonate salts, like K_2_CO_3_, in high concentrations to allow for sustainable and efficient carbon conversion rates. The high salinity and pH of the carbonate solution that circulates between the absorption and desorption column challenge the stability of biocatalysts, which, like all conventional proteins, tend to denature in strong alkaline media. To test this crucial parameter for a candidate CO_2_ capture enzyme, CA-KR1 was incubated in 20% (w/v) aqueous K_2_CO_3_ (pH 11.5) for up to 30 d, and its residual solubility was measured as an indication of alkali-stability. Furthermore, to link the remaining solubility to activity, protonography tests were performed in parallel. Strikingly, the residual concentration of CA-KR1, as well as the protonography activity tests, showed that CA-KR1 retains 90% of its initial concentration even after 30 d of incubation while remaining active (Figure 2).

To the best of our knowledge, CA-KR1 is the first CA studied for solubility retention under long-term incubation in a 20% (w/v) K_2_CO_3_ solution, which is the standard solvent for HPC. Typically, the alkali-stability of CAs is evaluated using glycine or NaOH-based buffering systems, which are not relevant to this application. Examples of such enzymes include SazCA and SspCA, with a pH optimum of 9.6.^17^ Other highly alkali-stable CAs include ApCA, which retains 60% of its activity after 36 h at pH 11, and LdCA, which maintains approximately 30% of its initial activity after 30 min at the same pH.^80,81^ One of the most alkali-stable and recently studied CAs is BhCA, retaining 65 and 20% of its activity after 1 and 2 d of incubation at pH 11, respectively.^82,83^ Based on these data, CA-KR1 emerges as one of the most alkali-stable CAs in the context of HPC. Our findings, combined with the thermostability profile of CA-KR1, demonstrate the potential of this new CA to perform multiple CO_2_ absorption–desorption cycles in biomimetic carbon-capture setups under realistic HPC conditions. The results indicate that long-term incubation of CA-KR1 in 20% K_2_CO_3_ does not impact the enzyme’s solubility and activity, suggesting a robust storage-stability profile, which is crucial for enzymes intended for product translation. As discussed in Section 3.6, CA-KR1 exhibited outstanding CO_2_ sequestration capabilities, particularly under strongly alkaline conditions (pH 11.5) and in combination with an elevated temperature of 90 °C, underscoring its stability in alkaline environments.

### 3.5 Kinetic Studies of the CA-KR1-Catalyzed CO_2_ Hydration Reaction

**Figure 3:**
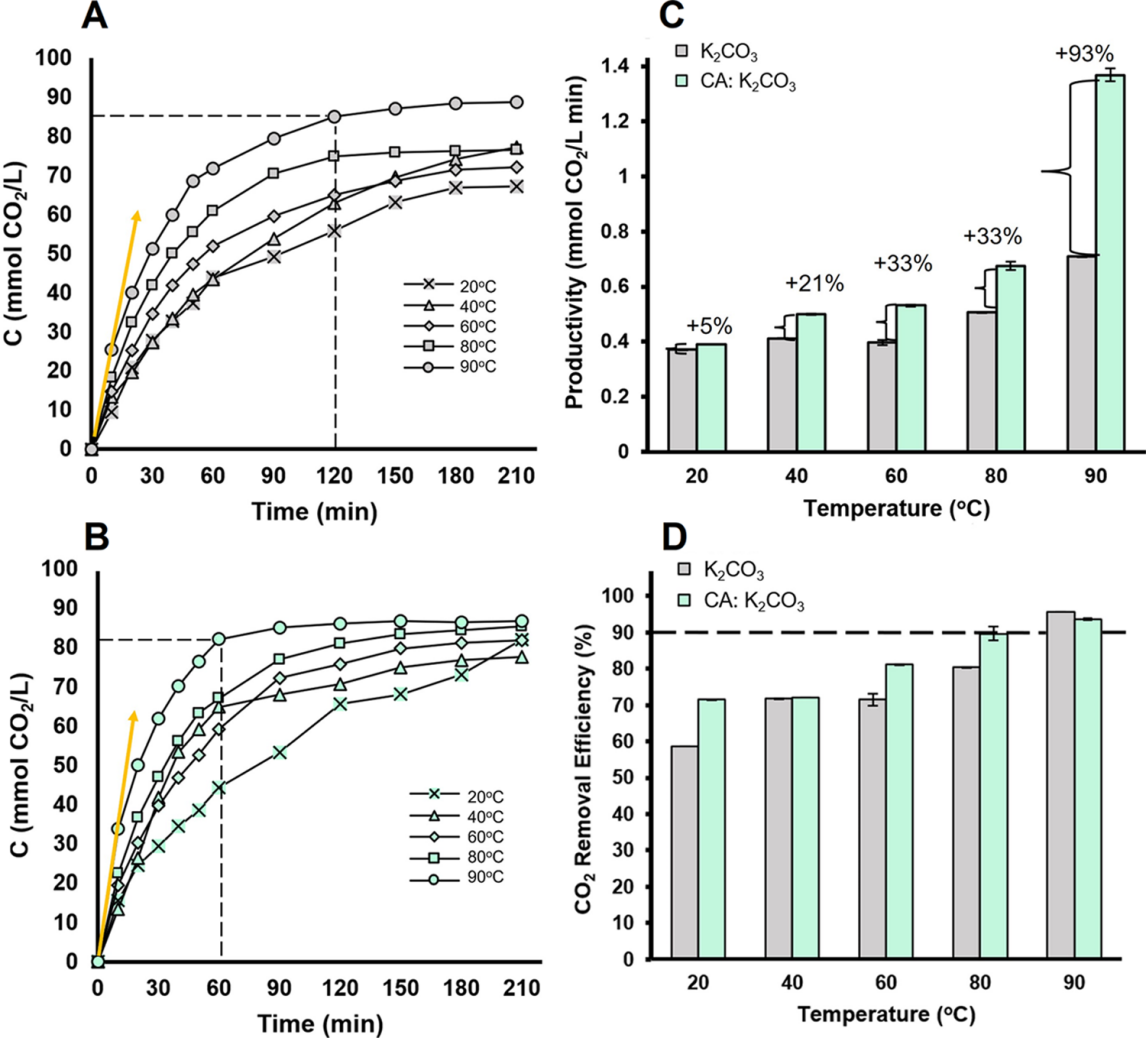
CA-KR1 performance as a promoter of CO_2_ capture in 20% K_2_CO_3_. (A) Concentration of CO_2_ absorbed over time during the CO_2_ capture reaction in 20% K_2_CO_3_ and (B) in 20% K_2_CO_3_ supplemented with CA-KR1. The yellow linear curve indicates the initial absorption rate at 90 °C, equal to 2.5 mmol of CO_2_/L min for 20% K_2_CO_3_ and 5.0 mmol of CO_2_/L min for CA-KR1:20% K_2_CO_3_. (C) Productivity is expressed as the total amount of CO_2_ absorbed per time of reaction until the reaction reaches a steady state and (D) CO_2_ removal efficiency. Capture from CO_2_-rich gas was evaluated in a pressurized batch reactor over a range of temperatures (20–90 °C). To start the reaction, the system was pressurized with gas (20% CO_2_) at 7 bar, and the pressure drop was monitored over time. All reactions were carried out in duplicate.

The method employed for the determination of the kinetic parameters of the CA-KR1 catalyzed CO_2_-hydration reaction was stopped-flow spectrophotometry at 25 °C. Table 1 presents the kinetic constants of CA-KR1, which follows Michaelis–Menten kinetics, as well as the kinetic constants of known CAs from different families. All experiments were performed at 25 °C, and the pH of the buffering system that was used had an initial value of 8.3. In this setup, CA-KR1 exhibited a catalytic turnover number (*k*_cat_) of 1.2 × 10^3^ s^–1^ and a *K*_M_ of 4.9 mM, resulting in a catalytic efficiency of 2.4 × 10^5^ M^–1^ × s^–1^. Although the determined *K*_M_ is within the range of reported values of known CAs, the *k*_cat_ value is low compared with other known bacterial CAs. This very likely occurs since CA-KR1 is a thermo- and alkali-stable enzyme, thus exhibiting decreased activity under low temperatures conditions. This hypothesis can be supported by the results in Section 3.6 (Figure 3), which clearly demonstrate that the enzyme exhibits very low activity at temperatures like those used in the kinetic experiments, in stark contrast to the high activity observed at 90 °C. Besides, pH and decreased temperature are known to have a minor effect on the affinity of the enzyme to the substrate (*K*_M_) but a major effect on the turnover rate of an enzyme (*k*_cat_), as raised temperature generally accelerates reactions. An example of pH-related behavior from the literature is CA CcaA274,^84^ where the authors suggest that the use of pH 9.5 buffer instead of pH 7.5 buffer results in a 20-fold increase in *k*_cat_. Furthermore, different temperature- and pH-dependent isoforms of β-CA enzymes are known to adapt different active site conformations. This ability to switch between different active sites suggests that the enzyme can adapt to varying environmental conditions, potentially allowing it to optimize its function depending on factors like pH or temperature.^85,86^ Thus, the kinetic values reported here are most likely an underestimation of the true performance potential of the new enzyme for industrial use since the reaction conditions used to define them differ dramatically from the conditions of industrial CO_2_ capture where the new biocatalyst potentiates to be implemented. The use of ambient conditions in our studies, as in the vast majority of the reported CA discovery studies, is due to limitations of the available lab-scale CA activity assays imposed by the gaseous nature of the substrate and the indirect measurement of CO_2_ conversion.^75^

**Table 1:** Kinetic Constants of CA-KR1 Catalyzed CO_2_ Hydration and Comparison to Reported Carbonic Anhydrases

CA	family	*k*_cat_ (s^–1^)	*K*_M_ (mM)	*k*_cat_/*K*_M_ (M^–1^ × s^–1^)	references
TaCA	α	1.6 × 10^6^	9.9	1.6 × 10^8^	(55)
SspCA	α	9.4 × 10^5^	8.4	1.1 × 10^8^	(87)
**CA-KR1**	**β**	**1.2 × 10**^**3**^	**4.9**	**2.4 × 10**^**5**^	**this work**
CcaA274 (pH 7.5)	β	3.34 × 10^3^	2.8	1.19 × 10^6^	(84)
CcaA274 (pH 9.5)	β	6.26 × 10^4^	15.8	3.96 × 10^7^	(84)
PgiCA	γ	4.1 × 10^5^	7.5	5.4 × 10^7^	(88)

### 3.6 Performance Evaluation of CA-KR1 under Realistic HPC CO_2_ Capture Conditions

Due to the restricted solubility of CO_2_ at elevated temperatures and the incompatibility of high pH solutions with the standard colorimetric CA assay, investigating thermostable and alkali-stable CAs under optimal conditions using conventional laboratory protocols and equipment is technically unfeasible. For this reason, the industrial potential of CA-KR1 was assessed by testing its performance in a CO_2_-capture batch reactor operating under high temperature and alkalinity conditions, a setup representing the most suitable lab-scale configuration for evaluating a novel CA enzyme. For this reason, the enzyme was used as a promoter in a CO_2_ capture trial. Within the batch reactor, a 20% K_2_CO_3_ aqueous solution (w/v) was supplemented with CO_2_-rich gas at an initial pressure of 7 bar. CO_2_ absorption was tested at different temperatures, including conditions relevant to the HPC technology (80–90 °C). The addition of CA-KR1 in the aqueous carbonate solution increased the overall productivity by a range of 5–33% between 20 and 80 °C. Importantly, an impressive 93% increase in CO_2_ absorption productivity was observed at 90 °C (Figure 3C). Interestingly, these findings further highlight the thermophilic nature of CA-KR1 while revealing that the enzyme remains active under a wide range of temperatures for several hours. At temperatures above 80 °C, CO_2_ removal efficiency higher than 80% was recorded (Figure 3D). Specifically, the incorporation of the enzyme improved the CO_2_ removal efficiency of the carbonate system to 90% at 80 °C, surpassing nonenzymatic HPC by 10% and meeting the feasibility cutoff at this temperature, as 90% CO_2_ removal is the implementation threshold for carbon-capture technologies. At 90 °C, the removal efficiency was above 90% and independent of the presence of the enzyme, a fact that highlights the well-recognized potential of HPC technology. However, the superiority of the enzyme-promoted system is underlined since it presents a considerably higher productivity, translating in faster CO_2_ removal. Impressively, the CA-KR1-promoted CO_2_ capture reaction reached a plateau at only 60 min of reaction at 90 °C compared to the nonenzymatic reaction that required double the time, 120 min, to reach the same CO_2_ removal range (Figure 3A,B). Additionally, the presence of CA-KR1 resulted in a 2-fold higher initial absorption rate compared to the nonenzymatic system. The remarkable CO_2_-sequestrating performance of CA-KR1 under high pressure and temperatures in strong alkaline solutions, combined with its high thermostability at 90 °C for at least 6 h of operation, renders it an unprecedentedly promising green promoter for industrial CO_2_ capture processes with focus on HPC. Our results clearly demonstrate the industrial significance of CA-KR1 and introduce it to the carbon-capture biocatalysts portfolio. Although the enzymatic concentration used herein was much lower than the 0.2–2 g/L range used in large-scale industrial BioHPC, the optimal concentration for the application cannot be extrapolated from this study. Ongoing studies in a pilot-scale BioHPC unit aim to determine the exact operation conditions for techno-economic optimization. Incorporating this novel enzyme in HPC setups aspires to enable absorber column downsizing and accommodation of faster sequestrating solvents. These improvements reduce capital costs, significantly enhancing the economic viability of carbon-capture processes and making them more accessible and attractive for widespread adoption across various industries.

### 3.7 Positioning of CA-KR1 in the Landscape of Carbon-Capture Innovation

In the era of the climate crisis, global research is focused on bridging the gap between the demand for carbon capture and the available solutions. A promising approach involves exploring robust carbonic anhydrases (CAs) for sustainable green biomimetic CO_2_ capture. Using an in-house-developed metagenomic data mining bioinformatics pipeline, we identified CA-KR1, a thermo-alkali-stable CA that performs exceptionally well under Hot Potassium Carbonate capture (HPC) conditions. This novel enzyme shows high thermostability and remarkable efficiency in a 20% K_2_CO_3_ aqueous solution, which is the standard medium for industrial HPC capture. CA-KR1 improves CO_2_ removal productivity by 93% at 90 °C compared to standard nonenzymatic HPC capture, a state-of-the-art technology. It also enables 90% CO_2_ removal at 80 °C, outperforming HPC removal by 10%, and doubles the initial CO_2_ absorption rate at 90 °C. The discovery of such potent promoters for green carbon-capture technologies is crucial for achieving carbon neutrality. The significance of these findings is underscored by the development of Bluezyme, a pioneering biomimetic CO_2_-capture solution introduced in 2023 by Saipem in collaboration with Novozymes.^89^ This milestone highlights the critical role of potent CA biocatalysts, such as CA-KR1, in shaping a diverse and effective biocatalyst portfolio for carbon capture and fostering innovation in this critical domain. CA-KR1’s unparalleled stability is expected to enable high-temperature carbon-capture processes, merging the benefits of HPC and enzyme-mediated capture. This makes CA-KR1 a leading candidate for BioHPC capture, with its deployment in industrial setups anticipated to significantly advance BioHPC capture technology. This transition from laboratory bench to bioreactor is a vital step toward the timely decarbonization of industrial processes.

## Supporting Information Available

Calculation of actual CO_2_ conversion rates (Text S1); calculation of CO_2_ hydratase activity values and residual (%) CO_2_ hydratase activity (Text S2); calculation of residual concentration (%) CO_2_ hydratase activity (Text S3); calculation of CO_2_ productivity and removal efficiency during HPC batch reactor experiment (Text S4); SDS-PAGE & CA activity detection of CA-KR1 (Figure S1); purification of recombinant CA-KR1 (Figure S2); and thermostability of CA-KR1 in comparison with the bibliography (Table S1) (PDF)
