# Supplementary material for: Biomimetic CO2 Capture Unlocked through Enzyme Mining: Discovery of a Highly Thermo- and Alkali-Stable Carbonic Anhydrase
Source: Environ Sci Technol. 2024 Sep 23;58(40):17732–42. doi: 10.1021/acs.est.4c04291 (PMC11465738; doi:10.1021/acs.est.4c04291)
Supplement: Supplementary file 1 — es4c04291_si_001.pdf [file es4c04291_si_001.pdf]

Supporting Information

Biomimetic CO_2 Capture Unlocked through Enzyme Mining: Discovery of a Highly Thermo- and Alkali-Stable Carbonic Anhydrase

Konstantinos Rigkos[1,2,3], Georgios Filis[1,3,4], Io Antonopoulou[5], Ayanne de Oliveira Maciel[5], Pavlos Saridis[1,6], Dimitra Zarafeta[1,3*], Georgios Skretas[1,3*]

[1]Institute of Chemical Biology, National Hellenic Research Foundation, Athens 11635, Greece

[2]Department of Biological Applications and Technologies, University of Ioannina, Ioannina 45500, Greece

[3]Institute for Bio-innovation, Biomedical Sciences Research Center "Alexander Fleming", Vari 16672, Greece

[4]Department of Informatics and Telecommunications, National and Kapodistrian University of Athens, Athens 16122, Greece

[5]Biochemical Process Engineering, of Division Chemical Engineering, Department of Civil, Environmental and Natural Resources Engineering, Luleå University of Technology, Luleå 97187, Sweden

[6]Faculty of Biology, National and Kapodistrian University of Athens, Athens 15772, Greece

*Correspondence: skretas@fleming.gr (Lead contact), zarafeta@fleming.gr

Number of pages: 10

Number of supporting texts: 4

Number of supporting figures: 2

Number of supporting tables: 1

Text S1. Calculation of actual CO_2 conversion rates

Text S2. Calculation of CO_2 hydratase activity values & Residual (%) CO_2 hydratase activity

Text S3. Calculation of Residual Concentration (%) CO_2 hydratase activity

Text S4. Calculation of CO_2 productivity and removal efficiency during HPC batch reactor experiment

Figure S1. SDS-PAGE & CA activity detection of CA-KR1

Figure S2. Purification of recombinant CA-KR1

Table S1. Thermostability of CA-KR1 in comparison with the bibliography

Text S1. Calculation of actual CO_2 conversion rates

The initial absorbance rates calculated during kinetic measurements were converted to actual CO_2 conversion rates through implementation of Eq. S1 as has been previously described [1].

$$\frac{dA}{dt} = \left(\frac{1}{Q}\right) \times \left(\frac{dx}{dt}\right) \qquad \textbf{(Eq. S1)}$$

Here the rate of absorbance change of the indicator phenol red at 557nm is presented as $\frac{dA}{dt}$, the rate of proton release as $\frac{dx}{dt}$, while Q represents the buffer factor.

Text S2. Calculation of CO_2 hydratase activity values & Residual (%) CO_2 hydratase activity

During thermostability studies of CA-KR1, the CO_2 hydratase activity of each sample was calculated in Wilbur-Anderson units (Eq. S2) [2].

$$WA = \frac{T0-T}{T} \qquad \textbf{(Eq. S2)}$$

Here the WA corresponds to the enzymatic CO_2 hydration activity expressed in Wilbur-Anderson units, T0 is the time of reaction without enzyme in seconds and T is time of reaction with the enzyme in seconds.

The Residual activity (%) for each thermally treated sample was calculated with the equation bellow (Eq. S3).

$$Residual\ activity\ (\%) = \left(\frac{WA\ thermally\ treated\ sample}{WA\ untreated\ sample}\right) \times 100 \qquad \textbf{(Eq. S3)}$$

Here the CO_2 hydration activity (expressed in Wilbur-Anderson units) of each thermally treated sample and for the control untread sample are presented as "WA thermally treated sample" and "WA untreated sample" respectively.

Text S3. Calculation of Residual Concentration (%) CO_2 hydratase activity

For the determination of the Residual concentration (%) of each alkali treated CA-KR1 sample the following equation was employed (Eq. S4)

$$Residual\ concentration\ (\%) = \left(\frac{[treated\ sample]}{[untreated\ sample]}\right) \times 100 \qquad \textbf{(Eq. S4)}$$

Here the concentration of each alkali treated sample and for the control untread sample are presented as "[treated sample]" and "[untreated sample]" respectively.

Text S4. Calculation of CO_2 productivity and removal efficiency during HPC batch reactor experiment

The amount of CO_2 absorbed during the reaction was calculated based on the ideal gas law and with the hypothesis that only CO_2 is absorbed in the liquid.

At t=0:
P= 7 bar, Gas phase: 20% CO_2 and 80% N_2, reactor head space: 0.1 L, T=20-90°C, depending on the experiment. At the gas phase of the reactor:

$$n_{total} = n_{N2i} + n_{CO2i} = \frac{P \cdot V}{R \cdot T} \qquad \textbf{(Eq. S5)}$$

$$n_{N2i} = 0.8 \cdot n_{total} \qquad \textbf{(Eq. S6)}$$

$$n_{CO2i} = 0.2 \cdot n_{total} \qquad \textbf{(Eq. S7)}$$

Were n_{total} represents the total amount of gas in moles while n_{N2i} and n_{CO2i} represent the initial amount of N_2 and CO_2 gas in moles respectively.

During the CO_2 absorption reaction, there is a pressure drop over time. The pressure drop is dependent on the amount of CO_2 that is getting absorbed in the liquid. Only CO_2 absorbed in the liquid. At the gas phase of the reactor:

$$n_{N2t} = n_{N2i} = constant \quad \textbf{(Eq. S8)}$$

$$n_{CO2t} = \frac{P(t) \cdot V}{R \cdot T} - n_{N2t} \quad \textbf{(Eq. S9)}$$

Were n_{N2t} and n_{CO2t} represent the total amount of N_2 and CO_2 gas in moles respectively in the head space at a specific time point t.

The amount of absorbed CO_2 in the liquid is calculated as:

$$n_{CO2abs} = n_{CO2i} - n_{CO2t} \quad \textbf{(Eq. S10)}$$

Were the CO_2 absorbed is presented as $n_{CO2abs.}$

The absorption productivity is defined as the mmol CO_2 absorbed per L absorbent per min. The time of reaction is defined as the point when the amount of CO_2 absorbed reaches a plateau.

$$Productivity = \frac{n_{CO2abs}}{V_{abs} \cdot t} \quad \textbf{(Eq. S11)}$$

The CO_2 removal efficiency is defined as the total amount of CO_2 that was absorbed divided by the total amount of CO_2 that was supplied in the reactor.

$$CO_2 \ removal \ efficiency \ (\%) = \frac{n_{CO2abs}}{n_{CO2i}} \quad \textbf{(Eq. S12)}$$

Figures

Figure S1

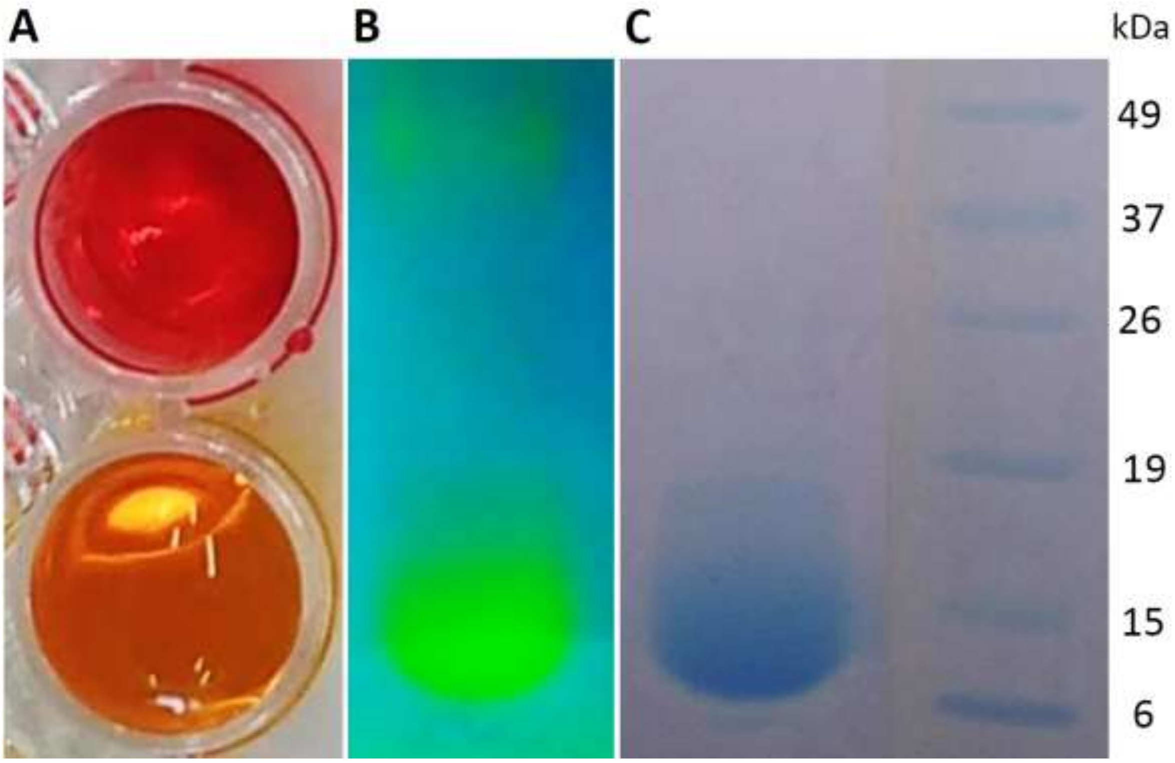

Figure S1: SDS-PAGE & CA activity detection of CA-KR1. **(A)** Wilbur-Anderson assay for the detection of CA activity. CAs catalyze the CO_2 hydration reaction: $CO_2 + H_2O \rightleftharpoons H_2CO_3 \rightleftharpoons HCO_3^- + H^+$. Proton production induces a pH drop, which can be visualized through the color change of a pH indicator. Two reactions containing saturated CO_2 water and phenol red (pH indicator) were prepared. In the reaction where the protein CA-KR1 was added (bottom well) the color change from red to yellow occurred immediately, whereas in the control reaction (upper well), where no protein was added, the change of color was delayed. The picture represents a 5 s snapshot of the experiment. **(B)** Protonography analysis of CA-KR1. Unboiled sample of purified CA-KR1 protein was analyzed by SDS-PAGE using a 15% gel. The gel was stained with the

pH indicator bromothymol blue. After immersion of the gel in CO_2-saturated water, the appearance of yellow bands occurs due to local drop in pH indicating CA activity. **(C)** Unboiled sample of purified CA-KR1 protein was analyzed by SDS-PAGE. The gel was stained, and the protein was visualized with Coomassie Blue. lane 1: CA-KR1 (predicted molecular weight: 19 kDa); Lane 2: molecular weight marker.

Figure S2

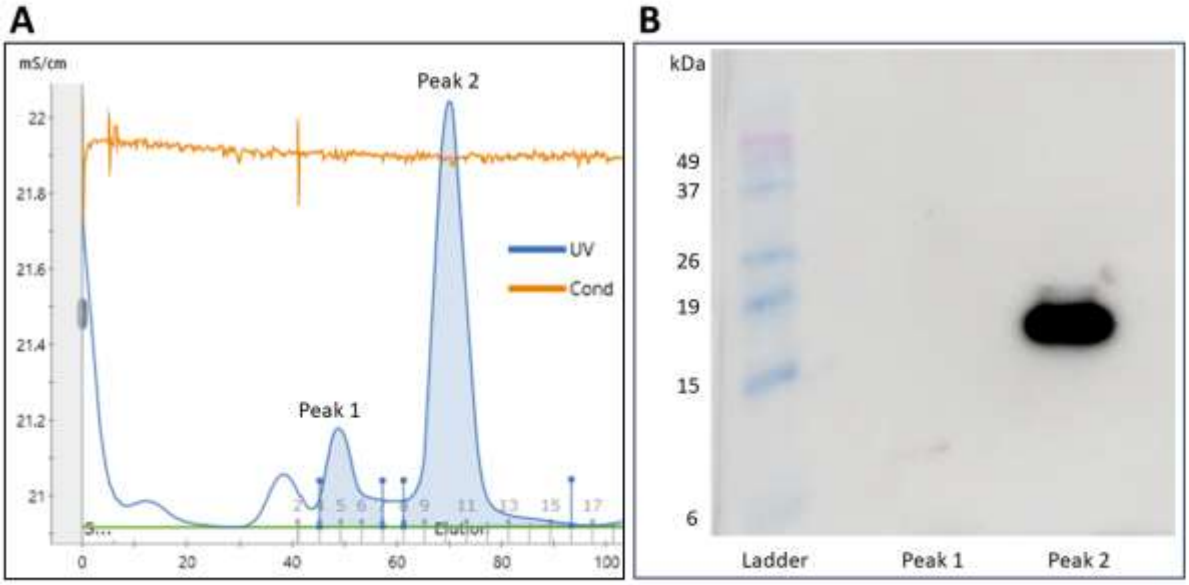

Figure S2: Purification of recombinant CA-KR1. **(A)** Size exclusion chromatography (SEC) profile (Peak 2 corresponds to the dimeric CA-KR1 at 38 kDa). **(B)** Western blot analysis (anti-polyHis antibody) of SEC fractions corresponding to peak 1 and peak 2 of the chromatograph.

Table S1. Thermostability of CA-KR1 in comparison with the bibliography

Carbonic Anhydrase	Class	Type	70°C		75°C	80°C			85°C		90°C				Reference
			1h	24h	8h	15min	1h	3h	8h	24h	15min	1h	2h	3h	
**CA-KR1**	**β**	**Wild type**	**112%**	**89%**	**-**	**-**	**87%**	**101%**	**-**	**-**	**-**	**89%**	**80%**	**77%**	**This work**
oTaCA	α	Wild type	~90%	-	-	-	~65%	-	-	-	-	~35%	-	-	[2]
TaCA	α	Wild type	~60%	-	-	-	~17%	-	-	-	-	0%	-	-	[2]
SspCA	α	Wild type	-	~55%	~15%	-	-	~76%	0%	-	-	-	-	~71%	[3-5]
SspCA (6M1)	α	Mutant	-	-	~50%	-	-	-	~25%	-	-	-	-	-	[5]
TaCA (N140G)	α	Mutant	100%	-	-	-	100%	-	-	-	-	100%	-	-	[6]
TaCA (S8R/G9P/ E22P)	α	Mutant	-	-	-	-	-	-	-	~73%	-	-	-	-	[5]
TaCA (SEQ ID NO: 6)	α	Mutant	-	~70%	-	-	-	-	-	~19%	-	-	-	-	[5]
PMCA (sp-)	α	Wild type	-	-	-	-	-	-	-	-	-	-	~75%	-	[7]
SazCA	α	Wild type	-	-	-	-	-	~42%	-	-	-	-	-	~29%	[8]
Cab	β	Wild type	-	-	-	~72%	-	-	-	-	~27%	-	-	-	[9]

References

1. Khalifah, R. G. The carbon dioxide hydration activity of carbonic anhydrase: I. Stop-flow kinetic studies on the native human isoenzymes B and C. *J. Biol. Chem.* **1971,** *246*, (8), 2561-2573.

2. James, P.; Isupov, M. N.; Sayer, C.; Saneei, V.; Berg, S.; Lioliou, M.; Kotlar, H. K.; Littlechild, J. A. The structure of a tetrameric α-carbonic anhydrase from Thermovibrio ammonificans reveals a core formed around intermolecular disulfides that contribute to its thermostability. *Acta Crystallogr., Sect. D: Biol. Crystallogr.* **2014,** *70*, (10), 2607-2618.

3. Del Prete, S.; Merlo, R.; Valenti, A.; Mattossovich, R.; Rossi, M.; Carginale, V.; Supuran, C. T.; Perugino, G.; Capasso, C., Thermostability enhancement of the α-carbonic anhydrase from Sulfurihydrogenibium yellowstonense by using the anchoring-and-self-labelling-protein-tag system (ASL tag). *Journal of enzyme inhibition and medicinal chemistry* **2019,** *34*, (1), 946-954.

4. Capasso, C.; De Luca, V.; Carginale, V.; Cannio, R.; Rossi, M. Biochemical properties of a novel and highly thermostable bacterial alpha-carbonic anhydrase from Sulfurihydrogenibium yellowstonense YO3AOP1. *J. Enzyme Inhib. Med. Chem.* **2012,** *27*, (6), 892-897.

5. Voyer, N.; Daigle, R.; Madore, É.; Fradette, S. VARIANTS OF THERMOVIBRIO AMMONIFICANS CARBONIC ANHYDRASE AND CO_2 CAPTURE METHODS USING THERMOVIBRIO AMMONIFICANS CARBONIC ANHYDRASE VARIANTS. US 10 415 028 B2, **2019**.

6. Parra-Cruz, R.; Lau, P. L.; Loh, H.-S.; Pordea, A. Engineering of Thermovibrio ammonificans carbonic anhydrase mutants with increased thermostability. *J. CO2 Util.* **2020,** *37*, 1-8.

7. Kanth, B. K.; Jun, S.-Y.; Kumari, S.; Pack, S. P., Highly thermostable carbonic anhydrase from Persephonella marina EX-H1: its expression and characterization for CO2-sequestration applications. *Process Biochemistry* **2014,** *49*, (12), 2114-2121.

8. De Luca, V.; Vullo, D.; Scozzafava, A.; Carginale, V.; Rossi, M.; Supuran, C. T.; Capasso, C., An α-carbonic anhydrase from the thermophilic bacterium Sulphurihydrogenibium azorense is the fastest enzyme known for the CO2 hydration reaction. *Bioorganic & medicinal chemistry* **2013,** *21*, (6), 1465-1469.

9. Smith, K. S.; Ferry, J. G. A plant-type (beta-class) carbonic anhydrase in the thermophilic methanoarchaeon Methanobacterium thermoautotrophicum. *J. Bacteriol.* **1999,** *181*, (20), 6247-6253.
